# Chronic Fluoxetine Treatment Induces Brain Region-Specific Upregulation of Genes Associated with BDNF-Induced Long-Term Potentiation

**DOI:** 10.1155/2007/26496

**Published:** 2007-09-19

**Authors:** Maria Nordheim Alme, Karin Wibrand, Grethe Dagestad, Clive R. Bramham

**Affiliations:** Department of Biomedicine and Bergen Mental Health Research Center, University of Bergen, Jonas Lies vei 91, 5009 Bergen, Norway

## Abstract

Several lines of evidence implicate BDNF in the pathogenesis of stress-induced depression and the delayed efficacy of antidepressant drugs. Antidepressant-induced upregulation of BDNF signaling is thought to promote adaptive neuronal plasticity through effects on gene expression, but the effector genes downstream of BDNF has not been identified. Local infusion of BDNF into the dentate gyrus induces a long-term potentiation (BDNF-LTP) of synaptic transmission that requires upregulation of the immediate early gene Arc. Recently, we identified five genes (neuritin, Narp, TIEG1, Carp, and Arl4d) that are coupregulated with Arc during BDNF-LTP. Here, we examined the expression of these genes in the dentate gyrus, hippocampus proper, and prefrontal cortex after antidepressant treatment. We show that chronic, but not acute, fluoxetine administration leads to upregulation of these BDNF-LTP-associated genes in a brain region-specific pattern. These findings link chronic effects of antidepressant treatment to molecular mechanisms underlying BDNF-induced synaptic plasticity.

## 1. INTRODUCTION

Stable activity-dependent changes in synaptic efficacy, as seen during long-term potentiation (LTP) and long-term depression (LTD), require new gene expression. Persistent changes of this kind are thought to underlie long-term adaptive responses in behaviour, including memory formation, motivation, mood, and pain control. Maintenance of LTP induced by high-frequency stimulation (HFS) of excitatory glutamatergic synapses is typically divided into a transient early phase and a persistent late phase [1]. Development of late LTP requires gene transcription and protein synthesis, a process referred to as LTP consolidation. One of the major regulators of LTP consolidation is the secretory polypeptide brain-derived neurotrophic factor (BDNF) [2, 3]. BDNF is released postsynaptically from or near glutamate synapses in response to HFS and signals through TrkB receptor tyrosine kinases located pre- and post-synaptically [4–7]. Exogenous application of BDNF induces a long-term potentiation (BDNF-LTP) that mimics the late phase of LTP [8, 9]. In the dentate gyrus of adult rats in vivo, BDNF-LTP induction requires extracellular signal-regulated kinase activation, new gene transcription, and is occluded by prior expression of late LTP [9, 10] BDNF-LTP is associated with rapid upregulation and dendritic transport of mRNA encoded by the immediate early gene, activity-regulated cytoskeleton-associated protein (Arc, aka Arg3.1). Furthermore, Arc synthesis is necessary for the consolidation of BDNF-LTP and HFS-induced LTP [11, 12].

Several lines of evidence from human and animal studies implicate BDNF in the pathogenesis of stress-induced depression and the delayed efficacy of antidepressant drugs [13–16]. Rodent studies suggest that stress exposure reduces BDNF expression and TrkB signaling in the hippocampal formation and neocortex, while antidepressant treatment increases TrkB signaling and counteracts the behavioral effects of stress [16–20]. It has been suggested that antidepressant-induced upregulation of BDNF signaling promotes activity-dependent synaptic plasticity, synaptic reorganization, and neuronal survival [14–21]. Such adaptive responses require new gene expression, but the effector genes downstream of BDNF has not been identified.

In a recent microarray screen, we identified five genes that are strongly coupregulated with Arc mRNA during both BDNF-LTP and HFS-LTP in the dentate gyrus [22]. These genes are neuritin, neuronal activity-regulated pentraxin (Narp), TGF*β*-inducible early gene (TIEG1), calcium/calmodulin kinase-related peptide (Carp), and ADP-ribosylation factor-like protein-4 (Arl4d). Briefly, neuritin, also known as candidate platicity gene 15 (CPG15), is an immediate early gene which encodes a small, extracellular glycosylphosphatidylinositol (GPI)-anchored protein important for dendritic outgrowth and maturation [23, 24]. Narp is an immediate early gene in the pentraxin family of secreted lectins. Narp forms pentraxin assemblies that contribute to both activity-dependent and activity-independent excitatory synaptogenesis [25]. TIEG1, a member of the specificity protein/Kruppel-like factor (SP/KLF) family of zinc-finger transcription factors [26, 27], enhances TGF-*β*-dependent gene expression [28]. Carp, also known as ANIA-4 [29], contains the auto-inhibitory domain of CaMK-IV, suggesting that it may modulate CaM-kinase activity [30]. ADP-ribosylation factors (ARFs) and ARF-like proteins (ARLs) belong to the Ras superfamily of small GTP-binding proteins (GTPases) involved broadly in intratracellular trafficking and signaling [30, 32]. The rat gene Arl4d is a predicted sequence from the human gene ARL4D (former name ARF4L); new nomenclature described in [33].

Because of a possible link between BDNF-mediated effects of antidepressant treatment and synaptic plasticity, these BDNF-LTP coupled genes are all attractive as possible effectors of chronic antidepressant treatment. Here, we demonstrate brain region-specific upregulation of BDNF-LTP associated genes (neuritin, Narp, TIEG1 Arl4d, Carp, and Arc) after chronic, but not acute, fluoxetine administration. These findings suggest that antidepressant treatment promotes gene expression responses linked to BDNF signaling and long-term synaptic plasticity.

## 2. EXPERIMENTAL PROCEDURES

These experiments were approved by the Norwegian Committee for Animal Research in accordance with European Community Council Directives.

### 2.1. Animals

Adult male Spraque-Dawley rats (Møllegårds Avlslaboratorium, Denmark) weighing 190–300 g were housed in groups of four in a climate controlled room of constant temperature (21±1°C) and humidity. They were kept on a 12-hour light/dark cycle (lights on at 7:00 AM, lights off at 7:00 PM). Food and water were available ad libitum. For the acute treatments, rats received a single intraperitoneal (IP) injection of fluoxetine (10 mg/kg) or saline (0.9% NaCl) at 10:00 AM on the day of the experiment. For the chronic treatments, rats received daily (at 10:00 AM) IP injections of fluoxetine (10 mg/kg/day) or saline (0.9% NaCl) for 21 days. Two hours after the last injection the rats were anesthetized (urethane, 25 mg/kg IP), decapitated, and the brain was removed. The 2-hour time point was chosen because of previous evidence that translation factors are modulated at this time and because the mRNAs under study are all induced within 1–2 hours of BDNF infusion into the dentate gyrus [22, 34]. The dentate gyrus, hippocampus proper, and prefrontal cortex were rapidly dissected on ice and kept cold by repeated rinses in ice-cold artificial cerebrospinalfluid, ACSF (in mM): NaHCO_3_ (26), NaCl (124), KCl (3), MgSO_4_ (1.3), NaH_2_PO_4_ (1.25), glucose (10), pH (7.4). The prefrontal cortex was removed first. The dentate gyrus and the hippocampus proper were separated by unfolding the dentate gyrus from the hippocampus, leaving the CA1 and CA3 regions exposed. The tissue was rapidly frozen on a dry-ice/ethanol slurry and stored at −80°C.

### 2.2. Poly(A) RNA and cDNA preparation

Poly(A) RNA was isolated using the Dynabeads mRNA direct kit (Dynal, Oslo, Norway) according to the manufacturer's protocol. Minor modifications were that 70 *μ*L magnetic beads were used per sample and that the isolated poly(A) RNA fraction was eluted in 3×30 *μ*L elution buffer. The yield and quality of the poly(A) RNA were determined by measuring the absorbance at 260/280 nm. 60 ng poly(A)RNA was reversed transcribed using the Superscript First-Strand Synthesis Kit (Invitrogen). The cDNA was diluted 20-fold.

### 2.3. Semiquantitative real-time PCR and normalization strategies

Semiquantitative real-time PCR was performed on an iCycler (Bio-Rad) using cDNA from individual animals and the iQ SYBR Green Supermix. Five *μ*L cDNA was added to the PCR reaction mix to yield a total of 25 *μ*L. PCR quantification was performed in triplicate and the fluorescence signal was quantified by the second derivative maximum method $2^{-\Delta \text{Ct}}$, where $\Delta \text{Ct}={\text{Ct}}_{\text{gene}}-{\text{Ct}}_{\text{housekeeping gene}}$ for each animal using the iCycler iQ real-time detection system software. Student's t-test was used for the statistical analyses. Primers used are given in Table 1.

The determination of an appropriate housekeeping gene is discussed in several papers [35]. Five housekeeping genes have been analyzed for this purpose: Gadph, tubulin, Arp, cyclopholine, and polyubiquitine. gNorm was used for stability analyses and polyubiquitine was chosen as the preferred housekeeping gene. Primer sequences in 5′ to 3′ direction and annealing temperatures are given in Table 1.

## 3. RESULTS

### 3.1. Chronic fluoxetine treatment leads to brain region-specific upregulation of genes coupled to BDNF-LTP

Rats received acute (1 day) or chronic (21 days) treatment with the selective serotonin reuptake inhibitor, fluoxetine. Semiquantitative real-time PCR was used to determine changes in the expression of BDNF-LTP coupled genes in microdissected prefrontal cortex, hippocampus proper, and dentate gyrus. These brain regions were selected for analysis because of their role in memory loss and other cognitive features of affective disorders, and because chronic antidepressant treatment is thought to upregulate BDNF function in these regions [13].

**Figure 1:**
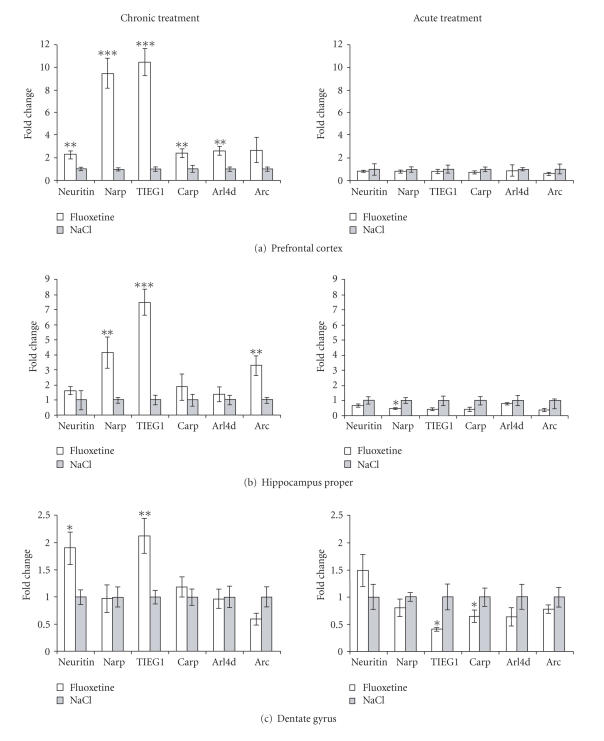
Chronic fluoxetine administration leads to brain region-specific upregulation of LTP-associated genes. Changes in mRNA expression following fluoxetine treatment are expressed as fold change relative to the saline control group. (a) Prefrontal cortex, (b) hippocampus proper, (c) dentate gyrus. N=7 fluoxetine chronic, n=8 NaCl chronic, n=5 fluoxetine acute, n=4 NaCl acute (except neuritin chronic NaCl in DG, n=7). *P<.05, **P<.01, **P<.001 from Student's t-test.

In prefrontal cortex (Figure 1(a)), mRNA levels for Narp and TIEG1 were significantly upregulated more than 9-fold after chronic fluoxetine administration, relative to saline control. Neuritin, Carp, and Arl4d were significantly elevated more than 2-fold in response to chronic fluoxetine treatment. In contrast, none of the mRNAs examined showed altered expression following acute fluoxetine treatment.

In hippocampus proper (Figure 1(b)), TIEG1 mRNA levels were elevated 7-fold, Narp was elevated 4-fold, and Arc was elevated 3-fold following chronic antidepressant treatment. In the acute fluoxetine group, Narp expression was reduced while expression of the remaining BDNF-LTP linked genes was unchanged.

In the dentate gyrus (Figure 1(c)), neuritin and TIEG1 were significantly upregulated 2-fold in the chronic fluoxetine group. Acute fluoxetine treatment produced a significant decline in TIEG1 and Carp mRNA expression.

### 3.2. Chronic fluoxetine treatment leads to brain region-specific regulation of BDNF exon-III specific mRNA

The BDNF gene consists of four 5′ noncoding exons (I-IV) each with a separate promotor and one 3′ exon (exon-V) encoding the mature BDNF protein. The exon-III and IV BDNF promoters are regulated as immediate early genes [36, 37]. Previous work showed that BDNF-LTP is associated with rapid upregulation of exon-III specific and exon-V (total) BDNF mRNA [22]. We examined expression of these transcripts in order to further compare the effects of fluoxetine treatment with that of BDNF infusion.

**Figure 2:**
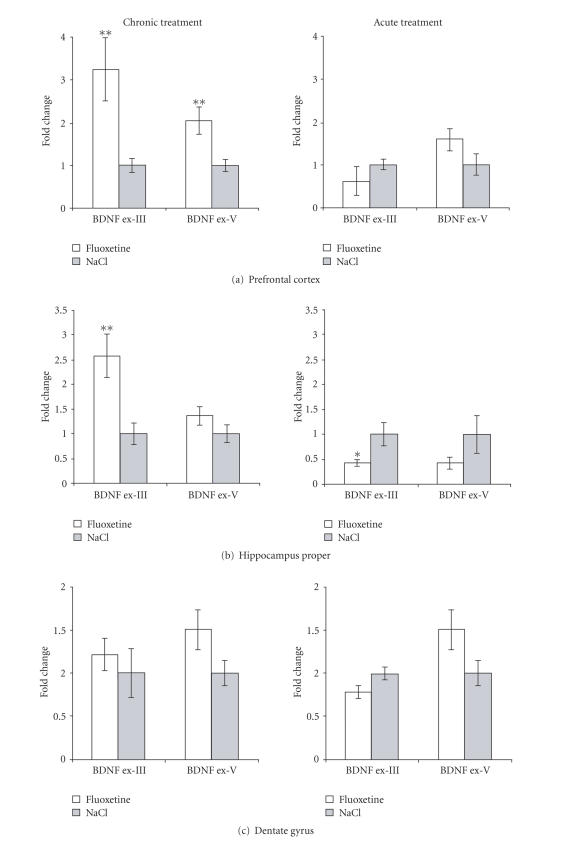
Brain region-specific upregulation of BDNF exon-III specific mRNA in chronic fluoxetine-treated rats. (a) Prefrontal cortex, (b) hippocampus proper, (c) dentate gyrus. N=7 fluoxetine chronic, n=8 NaCl chronic, n=5 fluoxetine acute, n=4 NaCl acute. *P<.05, **P<.01 Student's t-test.

In prefrontal cortex (Figure 2(a)), exon-III and exon-V BDNF mRNA levels were significantly elevated about 3-and 2-fold, respectively, after chronic antidepressant treatment. No significant changes in prefrontal cortex BDNF expression were observed following acute treatment. In hippocampus proper, chronic fluoxetine administration led to a 2.5-fold increase in exon-III expression, but no significant change in exon-V expression (Figure 2(b)). In contrast, acute treatment produced a significant decrease in exon-III BDNF mRNA levels relative to saline-injected controls. In the dentate gyrus, fluoxetine treatment had no significant effects on exon-III or exon-V BDNF mRNA expression at the times examined (Figure 2(c)).

## 4. DISCUSSION

This study demonstrates that chronic, but not acute, administration of fluoxetine leads to brain-region specific upregulation of genes associated with BDNF-induced LTP and HFS-induced LTP in the adult brain. This finding supports the idea that chronically administered antidepressants promote BDNF-induced gene expression and synaptic plasticity in multiple brain regions. The study also identifies neuritin, Narp, TIEG1, Carp, and Arl4d as candidate effectors of chronic fluoxetine treatment.

These five genes were originally identified in a microarray screen for mRNAs that are co-upregulated with Arc during long-term synaptic plasticity. Using in situ hybridization, Wibrand et al. [22] confirmed enhanced mRNA expression for all genes in dentate granule cells following both BDNF-LTP and HFS-LTP. The kinetics of mRNA expression was rapid (40 minutes post-HFS) and sustained (3 hours post-BDNF). In chronic fluoxetine-treated rats, all genes were upregulated in at least one of three brain regions studied (prefrontal cortex, dentate gyrus, and hippocampus proper), and each brain region exhibited a unique gene expression pattern. In the prefrontal cortex, neuritin, Narp, TIEG1, Carp, and Arl4d were all strongly upregulated. In the hippocampus proper, neuritin, Narp, TIEG1, and Arc were upregulated. In the dentate gyrus, only neuritin and TIEG1 were upregulated after three weeks of fluoxetine treatment. The basis of the regional effects on gene expression is unclear at present. Regional differences in the time course of TrkB signaling is one possible explanation. A single injection of fluoxetine, although sufficient to activate TrkB receptors [20], does not induce enhanced expression of BDNF-LTP-coupled genes. Repetitive treatment may produce regional patterns of BDNF-TrkB signaling and gene expression that are not seen following acute treatment. Regional gene expression may also be strongly gated by serotonin and by fluoxetine-induced modulation of trophic factors such as activin [38]. Further studies are needed to resolve the detailed time course of gene expression during chronic antidepressant treatment as well as the duration of gene expression following cessation of treatment.

Although acute fluoxetine did not enhance gene expression in any of the brain regions studied, Narp expression was significantly decreased in the hippocampus, while TIEG1 and Carp expression was decreased in the dentate gyrus. The discussion below concentrates on responses specific to chronic antidepressant regimens, yet it is important to bear in mind that acute effects may shed light on how the late effects develop. For example, rapid translation and degradation of preexisting mRNA in response to the first exposure to fluoxetine may be followed by a slow wave of new gene expression.

TIEG1 was the only gene upregulated in all brain regions analyzed. TIEG1 is an immediate early gene belonging to the specificity protein/Kruppel-like factor family of transcription factors. These transcription factors bind to GC-rich elements in the promoter region of target genes and are known to regulate a large number of genes involved in cell growth, differentiation and apoptosis [26, 39, 40]. TIEG1 is rapidly induced by TGF-*β*, a family of growth factors affecting gene expression and cellular functions primarily through activation of the Smad signaling pathways. TIEG1 potentiates the induction of TGF-*β*/Smad-regulated genes and overexpression of TIEG1 mimics the physiological effects of TGF-*β* in many cell types [28, 41]. In Aplysia, TGF-*β* induces a long-term increase in sensory neuron excitability and enhances transmission at sensorimotor synapses [42]. The pattern of upregulation of TIEG1 in the present study suggests that TGF-*β* signaling is widely upregulated following chronic fluoxetine treatment. Interestingly, phosphorylation of Smad2, one of the major mediators of TGF-*β* signalling, is also elevated following fluoxetine treatment [38]. In contrast to TIEG1, Smad2 phosphorylation is observed after both acute and chronic (3 week) treatment and only in the frontal cortex (not in hippocampus).

Narp and neuritin are suggested to play important roles in neuronal development and synapse formation. Narp induces AMPA receptor clustering and it has been shown that overexpression of Narp increases excitatory, but not inhibitory, synapse formation [43–45]. Neuritin is known to play important roles in synapse formation and maturation during development by promoting dendritic and axonal arbor growth [23, 24, 46, 47]. Neuritin is implicated in activity-dependent developmental plasticity in the cortex and transcription of neuritin in primary cortical neuron cultures is NMDA receptor-dependent and requires convergent activation of the CaMK and MAPK pathways [48, 49]. While the function of NARP and neuritin in adulthood is still little understood, upregulation of these genes implies effects on AMPA receptor clustering and synapse growth or reorganization.

Carp is one of several splice variants from the DCAMKL gene and contains 3 of its 15 exons. Carp lacks catalytic activity and is structurally similar to the autoinhibitory domain of CaMK-IV, a key regulator of calcium-induced gene expression [50, 51]. Previous studies have shown upregulation of Carp following kainic acid-induce seizures and it has been suggested that Carp is only expressed during cytotoxic conditions [30, 51]. This is in contrast to the results from the present study, where we show physiological expression of Carp in saline-injected controls and upregulation of expression in the prefrontal cortex following chronic fluoxetine treatment.

ADP-ribosylation factors (ARFs) and ARF-like proteins (ARLs) belong to the Ras superfamily of small GTP-binding proteins [31, 32]. The rat gene Arl4d is a predicted sequence from the humane gene ARL4D, a member of a subgroup of ARL-proteins characterized by the presence of a nuclear localization signal and rapid nucleotide exchange activity [52]. ARL GTPases have been suggested to serve as adaptors for cargoes lacking nuclear localization signals [53, 54]. In addition, Arl4d protein distributes to the plasma membrane when bound to GTP and to endosomes when bound to GDP, suggesting a role in transport between these compartments [54]. In situ hybridization shows that Arl4d mRNA is expressed in distinct nuclear punta in dentate granule cells [22]; a pattern reminiscent of nuclear structures such as PcG domains and coiled bodies. Chronic fluoxetine treatment increased the level of Arl4d mRNA about 2-fold in the prefrontal cortex, but did not change its expression in the hippocampus or dentate gyrus.

Arc mRNA was significantly upregulated 3-fold in the hippocampus proper, but was unchanged in the dentate gyrus. There was also a tendency for enhanced Arc expression in the prefrontal cortex (2.5-fold increase), though the interanimal variability was great. The present results appear consistent with earlier studies reporting enhanced Arc mRNA expression in the frontal cortex and CA1 region, but not in CA3 region or dentate gyrus, following treatment with the SSRI paroxetine either alone or in combination with antagonists of presynaptic ${\text{5-HT}}_{\text{1A}}$ receptors [55]. Chronic treatment with the triple monoamine reuptake inhibitor, tesofensine, similarly upregulated Arc in the CA1 region, but not in dentate gyrus [56]. Although the time course of gene induction needs to be resolved in finer detail, it is evident that Arc and the other BDNF-LTP genes are not always coexpressed.

Antidepressant-induced upregulation of total (exon-V) BDNF mRNA expression has been observed in many studies, but the regional expression pattern varies with the antidepressant used and treatment paradigm, with some studies reporting no effect (reviewed in [15, 16]). Recent studies suggest a similar variability in the effects of fluoxetine on exon-specific promoters. Dwivedi et al. [57] showed that chronic fluoxetine treatment (3 weeks of daily IP injection) had no effect on exon-III containing BDNF transcripts in the frontal cortex and hippocampus, yet increased exon-II BDNF mRNA levels in hippocampus. Another study reported upregulation of exon-I, but not exon-III, mRNA in the dentate gyrus and hippocampus after 3 weeks of fluoxetine administration [58]. Under the present conditions, total BDNF and BDNF exon-III were upregulated in the prefrontal cortex, while no significant effects were observed in the dentate gyrus. In hippocampus proper BDNF exon-III mRNA was upregulated, while no significant regulation of total BDNF was seen. We can only speculate on the reason for this discrepancy, which could reflect a larger fractional change in the expression of low abundance exon-III transcripts, or downregulation of transcription from other exon-specific promotors [58]. A similar strong upregulation of exon-III containing transcripts was previously observed following BDNF-LTP [22]. It should be emphasized, however, that the physiological impact of this BDNF mRNA upregulation remains unclear. Is it required for synaptic plasticity or does it reflect a delayed, compensatory resynthesis following BDNF release?

Several events associated with BDNF-LTP in the dentate gyrus are induced by chronic antidepressant treatment. Chronic antidepressant treatment leads to enhanced TrkB signaling, CREB activation [20, 59–61], and transcription of genes associated with BDNF-induced LTP. In addition to regulating transcription, BDNF modulates protein synthesis at the level of mRNA translation [12]. Thus, BDNF-LTP is associated with rapid and transient phosphorylation of two translation factors critical in controlling global translation initiation and elongation rates, eukaryotic initiation factor 4E (eIF4E), and elongation factor-2 (eEF2) [62]. Chronic fluoxetine-treatment similarly modulates eIF4E and eEF2 activity in the dentate gyrus [34]. Furthermore, acute infusion of BDNF into the dentate gyrus has behavioral antidepressant-like effects in rats [63]. Taken together, these data raises the possibility that antidepressants could work at least in part through molecular mechanisms similar to those seen during BDNF-LTP.

## Figures and Tables

**Table 1:** Primer sequences, annealing temperature, and accession numbers for the analyzed genes.

Gene	Primer sequence	Ann temp. (C°)	Acc number
Narp	Fw: GGCAAGATCAAGAAGACGTTG	58	P97738
	Bw: TCCAGGTGATGCAGATATGGT		
Carp	Fw: CAGGCAACCCTACCAGCATTT	58	AF030089
	Bw: TAACACTCCAACAGGCAGCA		
Arl4l	Fw: CTTCCCTTCTTTACCGCCTCA	58	XM_220933
	Bw: ACCCCCAACATCCCACACTT		
TIEG1	Fw: TAGTGTCTCAGTGCTCCGTCTG	62	NP_112397
	Bw: TGTGCTCCCTCTTTGGACTTTTC		
Neuritin	Fw: GGGACTTAAGTTGAACGGCA	56	NM_053346
	Bw: ACCCAGCTTGAGCAAACAGT		
BDNF exon-III	Fw: TGCGAGTATTACCTCCGCCAT	60	X67107
	Bw: AGGATGGTCATCACTCTTCTC		
BDNF exon-V	Fw: TGGGACTCTGGAGAGCGTGAATGG	62	BC087634
	Bw: CGGGACTTTCTCCAGGACTGTGAC		
Cyclopholine	Fw: AGCACTGGGGAGAAAGGATT	60	BC059141
	Bw: GATGCCAGGACCTGTATGCT		
Polyubiqutine	Fw: GGCAAGACCATCACCCTAGA	60	BC070919
	Bw: GCAGGGTTGACTCTTTCTGG		
Gapdh	Fw: CTGAGAATGGGAAGCTGGTCATC	62	DQ403053
	Bw: CAGTGGATGCAGGGATGATGTTC		
Tubulin	Fw: GGGAGCTCTACTGCCTGGAACATG	62	NM001007004
	Bw: GGAGACAATTTGGCTGATGAGGCG		
Arp	Fw: CGACCTGGAAGTCCAACTAC	60	NM022402
	Bw: ATCTGCTGCATCTGCTTG		
Arc	Fw: CCCAGTCTGTGGCTTTTGTCA	60	NM019361
	Bw: GTGTCAGCCCCAGCTCAATC		
